# An Atypical Case of Warfarin-Induced Skin Necrosis

**DOI:** 10.5811/cpcem.2017.3.33373

**Published:** 2017-10-11

**Authors:** Lindsay R. Sklar, Anne Messman

**Affiliations:** * Wayne State University, Department of Dermatology, Dearborn, Michigan; † Sinai-Grace Hospital, Department of Emergency Medicine, Detroit, Michigan

## Abstract

Skin necrosis is a relatively rare, potentially fatal side effect of warfarin. It is most commonly reported within 10 days of initiation of therapy in warfarin-naïve patients. We report an atypical case of warfarin-induced skin necrosis upon recommencement of warfarin in a non-naïve warfarin patient.

## INTRODUCTION

Warfarin is currently the most widely prescribed oral anticoagulant in North America. Thrombosis is a rare, paradoxical, and potentially fatal adverse effect of the drug. Skin necrosis occurs secondary to the development of microthrombi and endothelial cell damage in the vessels of dermal and subcutaneous tissues. Since this rare complication was first recognized in 1943, there have been an estimated 300 cases reported, affecting approximately 0.01–0.1% of patients on the drug.^1^ Typically, warfarin-induced skin necrosis presents within three to ten days of warfarin initiation in warfarin-naïve patients. Herein, we present an unusual case of warfarin-induced skin necrosis that presented upon the recommencement of warfarin in a patient who had previously been on the anticoagulant for two years without complication.

## CASE REPORT

**Image 1:**
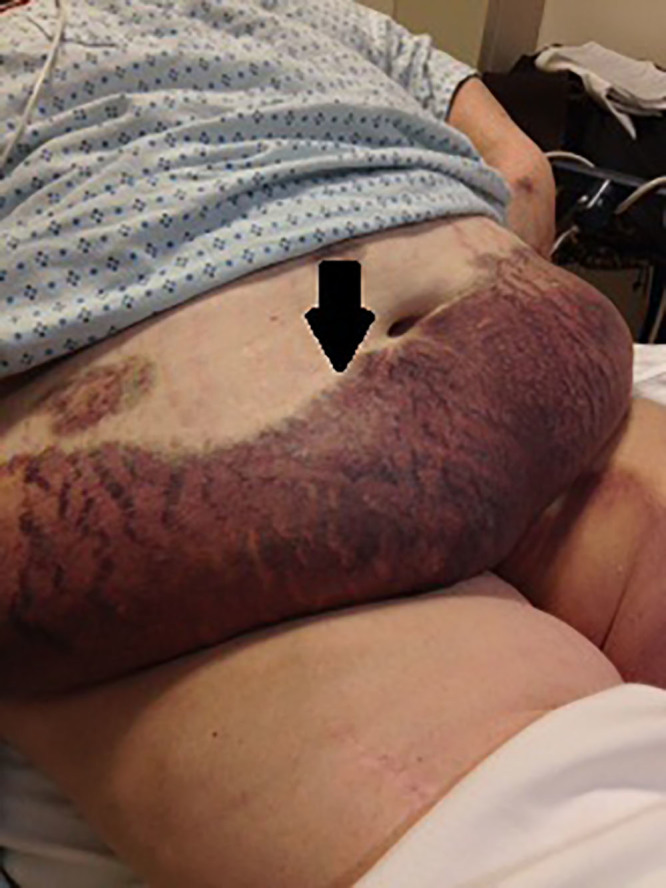
Ecchymoses secondary to warfarin-induced skin necrosis (arrow).

**Image 2:**
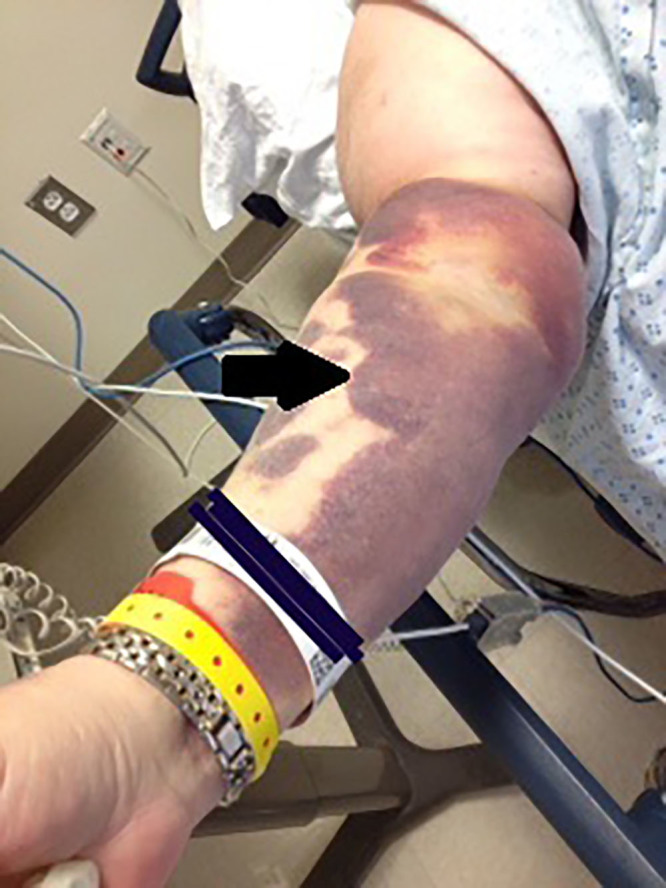
Ecchymoses secondary to warfarin-induced skin necrosis (arrow).

A 60-year-old morbidly obese Caucasian female with a past medical history of coronary artery disease, pulmonary embolism, and deep vein thrombosis presented to the emergency department (ED) complaining of excruciating pain associated with scattered ecchymoses. The patient’s vital signs were stable and within normal limits. Admission medications included warfarin 7.5 mg three days a week and 10 mg four days a week, aspirin 81 mg per day, and ticagrelin 90 mg per day. Physical examination revealed sharply demarcated, mildly indurated and excruciatingly tender, violaceous-to-black dusky patches with areas of necrosis overlying the left breast, pannus, right upper extremity, and left inner groin extending onto the left thigh (Images 1, 2). Laboratory studies were significant for a normal platelet level of 180,000/mm,^3^ partial thromboplastin time of 27.7 sec, prothrombin time of 21.8 sec, and an international normalized ratio (INR) of 1.87. Computerized tomography (CT) of the abdomen with and without intravenous contrast showed no evidence of malignancy or hemorrhage.

The patient had been on the same warfarin regimen (7.5mg three days a week and 10mg four days a week) for two years prior to presentation to our ED; it had been initiated after the development of a deep vein thrombosis and pulmonary embolism. She was compliant and maintained a therapeutic INR ranging from 2.3 to 2.8. Fifteen days prior to ED presentation, the patient underwent a cardiac catheterization, for which warfarin was held for a total of three days. The patient was anticoagulated with unfractionated heparin for the procedure. Upon recommencement of warfarin, the patient’s INR was sub-therapeutic, ranging from 1.7 to 1.8; enoxaparin was intermittently administered to achieve adequate anticoagulation. Six days after the catheterization, she developed atrial fibrillation, for which the warfarin dose was increased to 12.5 mg per day and the patient was placed on a continuous heparin infusion for three days. After day five of the 12.5 mg dose of warfarin and a continued sub-therapeutic INR, the patient noted minor left breast ecchymosis and tenderness. At that time, the warfarin dose was decreased to 7.5 mg per day. An ultrasound of the breast performed at this time to evaluate for a hematoma was normal. Within the subsequent five days, the benign-appearing ecchymosis had become unbearably tender, dark and dusky, and spread to involve the entire left breast, pannus, left upper extremity, and left inner groin and left thigh, which brought the patient to the ED.

With the possible diagnosis of warfarin-induced skin necrosis, warfarin was immediately discontinued. The patient was placed on a heparin continuous infusion and given vitamin K, four units of fresh frozen plasma, and started on rivaroxaban 20 mg per day. Her pain was managed with hydromorphone and acetaminophen-oxycodone. By day 11 of the admission, there was moderate improvement in both the clinical appearance and subjective tenderness of the affected skin.

## DISCUSSION

While the precise cause of warfarin-induced skin necrosis is heavily debated, it is agreed that the rapid decline of vitamin K-dependent coagulation factors with short half-lives, such as protein C or S, predisposes one to a temporary hypercoagulable state. Hypercoagulable states such as protein C or S deficiency, antithrombin III deficiency, factor V Leiden mutation, antiphospholipid antibody syndrome, and infectious states have all been described in association with warfarin-induced skin necrosis.^1^–^4^ The necrosis typically manifests as abrupt, painful, well-demarcated areas of ecchymosis, which can progress to hemorrhagic bullae within 24 hours. An eschar then forms, which eventually sloughs, revealing necrosis that may extend to the subcutaneous tissue.

Warfarin-induced skin necrosis most commonly affects obese, middle-aged women. Favored areas of involvement include those high in subcutaneous fat such as the abdomen, thighs, breasts, and buttocks. The condition most commonly presents within the first 10 days of warfarin initiation in warfarin-naïve patients, with the highest incidence occurring between days three to six. However, there are documented cases of warfarin-induced skin necrosis occurring months to years after the initiation of warfarin.^2^,^3^,^5^–^7^ Many of these late-onset cases involved patients who were predisposed to prothrombotic states.^2^,^3^,^8^,^9^ Furthermore, there have been three reported cases of warfarin-induced skin necrosis upon warfarin recommencement in warfarin non-naïve patients with no prior complications.^8^,^9^

**CPC-EM Capsule**

What do we already know about this clinical entity?

*Skin necrosis is a rare, potentially fatal side effect of warfarin that is most commonly reported within 10 days of initiation of therapy in warfarin-naïve patients.*

What makes this presentation of disease reportable?

*This is the fourth reported case of warfarin-induced skin necrosis in a warfarin non-naïve patient.*

What is the major learning point?

A prior history of being on warfarin without complication does not preclude warfarin-induced skin necrosis upon restarting warfarin in the future.

How might this improve emergency medicine practice?

There should be a high level of suspicion of warfarin-induced skin necrosis in all patients on warfarin presenting with skin tenderness and bruising.

O’Dempsey et al. reported the case of a male with a history of thrombophilia, factor V Leiden and prothrombin mutations who had been taking warfarin for 20 years without complication for thrombophilia. The warfarin was stopped for one day while he underwent repair of a ruptured aortic aneurysm. The patient developed warfarin-induced skin necrosis eight days after restarting warfarin, which was being administered with enoxaparin.^8^ Stewart et al. reported two patients who had been anticoagulated with warfarin without complication who then developed skin necrosis upon recommencement.^9^ The first patient was anticoagulated with warfarin for six months after developing a deep vein thrombosis. She developed another thrombus eight years later at which time warfarin was restarted; she developed warfarin-induced skin necrosis three days later. Laboratory investigation revealed lupus anticoagulant. The second patient reported by Stewart et al. was on warfarin for a history of pulmonary embolism. Upon becoming pregnant, she was anticoagulated with heparin alone and the warfarin was restarted after delivery. She developed warfarin-induced skin necrosis seven days later.

## CONCLUSION

The patient presented herein was on warfarin for two years; she discontinued warfarin for three days prior to recommencing the anticoagulant. She did experience a sudden increase in warfarin dosage, increasing from 7.5 mg to 12.5 mg shortly before presentation to the ED, which may offer a potential explanation for her presentation. Several authors argue that a large loading dose may predispose to warfarin-induced skin necrosis.^8^,^9^

The differential diagnosis of warfarin-induced skin necrosis includes heparin-induced thrombocytopenia, disseminated intravascular coagulation, purpura fulminans, necrotizing fasciitis, calciphylaxis, and cryoglobulinemia. It is crucial to take a careful history and physical examination in addition to ordering appropriate laboratory studies, as the timing of the onset of skin necrosis, the clinical clues upon exam, and pertinent laboratory findings often allow one to distinguish the true diagnosis. Biopsies are often non-diagnostic and are therefore not mandated in the diagnosis. This case should increase awareness that warfarin-induced skin necrosis can affect patients who are restarting warfarin, despite a history of chronic warfarin therapy without complication. In the ED, physicians must always have a high clinical suspicion for this rare, yet potentially fatal, reaction to warfarin. There should be a high level of awareness in all patients on warfarin presenting with skin tenderness and bruising, and physicians must have a low threshold for the immediate discontinuation of warfarin, initiation of heparin, administration of fresh frozen plasma, and vitamin K in these patients. Warfarin may be cautiously restarted at a low dose and gradually increased.
